# A new aerodynamic endonasal filtration technology for protection against pollutants and respiratory infectious agents: evaluation of the particle filtration efficacy

**DOI:** 10.3389/fmedt.2023.1219996

**Published:** 2023-07-21

**Authors:** Padmanabhan Saravanan, Francesco Broccolo, Nurshahidah Ali, Alden Toh, Sakinah Mulyana, Goh Lay Beng, Enrico Imperi, Alfredo Picano

**Affiliations:** ^1^ School of Applied Science, Temasek Polytechnic, Singapore, Singapore; ^2^ Department of Biological and Environmental Sciences and Technologies (DiSTeBA), University of Salento, Lecce, Italy; ^3^ Labor Industrial Research lab, Labor, Rome, Italy

**Keywords:** endonasal filters, air filtration, particulate matter, fine and ultrafine particles, pollutants

## Abstract

An innovative nasal filter was tested, based on aerodynamic air filtration and not on conventional air filtration by means of mesh filters. A custom testing system was designed and three sizes of the filter have been tested vs. monodispersed SiO_2_ particles sized 5 μm, 1 μm, and 0.5 μm under cycling flow of 6 liters per minute, provided by an artificial lung breather simulating spontaneous breathing. Accelerated testing was implemented, challenging filters with a maximum load of 200 mg per cubic meter. All three filters' sizes showed initial filtration efficiencies above 90% vs. all particles' sizes, decreased to not less than 80% after 30 min of accelerated testing, corresponding to 4.5 days of continuous use at 2 mg challenge, this value being associated with hazardous air conditions in the PSI scale. Results in this study indicate that nasal filters based on aerodynamic air filtration can provide fine and ultrafine filtration, offering protection in day-to-day life from risks associated with pollens, mites, PM, pollutants, and respiratory infectious agents, introducing acceptable respiratory resistance.

## 1. Introduction

Scientific and technological efforts have been devoted to the development of techniques to remove particles from the air, especially those smaller than 2.5 μm, which can cause serious health problems including respiratory allergies. Singapore's National Environment Agency (NEA) uses standards set by the United States Environmental Protection Agency (USEPA) to monitor air quality. The pollutant standards index (PSI) comprises six pollutants viz. sulphur dioxide (SO_2_), particulate matter (PM) [called "respirable suspended particles (RSP)" or PM10, as they are 10 microns or smaller in size], fine particulate matter (PM2.5), nitrogen dioxide (NO_2_), carbon monoxide (CO), and ozone (O_3_).

The unhealthy levels of fine particulate matter (PM2.5) have more serious health implications as they can penetrate the deeper regions of the respiratory tract, cause respiratory problems, and aggravate existing respiratory diseases. For each pollutant under PSI, a sub-index is calculated from a segmented linear function that transforms ambient concentrations onto a scale of 0–500 PSI; levels exceeding 300 are considered hazardous. The USEPA standard for 24-hour mean PM10 level and PM2.5 level is 150 μg/m^3^ and 15 μg/m^3^ respectively. The unhealthy range of PSI 300 and 400 values for RSP are 625 μg/m^3^ and 875 μg/m^3^ respectively. There are several different particles used in assessing respiratory gadgets performance viz. Al2O_3_, Fe_2_O_3_, SiO_2_, TiO_2_, NaCl, SWCNT, and diesel particulate matter (DPM) [using dioctyl phthalate (DOP), and oil] (1–3). Poly Alfa Olefin (PAO) or DOP/PAO produces mono or poly -dispersed test aerosol of sub-micron particles, generated to challenge (evaluate integrity) HEPA filters. Endonasal devices would help filter particulate matter in a hazardous range and provide protection to users on a day-to-day basis.

Filtration of ultrafine particles by means of fibrous filters is based on the requirement that the velocity of the flow through the filter is low enough (in the order of cm/s) to allow a sufficiently long residence time, to achieve a two-fold effect: allow interactions with the fibers to entrap finest particles and keep the pressure drop low in the presence of a tight mesh.

The endonasal application of mesh filters is highly demanding because it is heavily constrained by size. Each nostril is crossed by air at a speed of 1–1.5 m/s (4), a hundredfold that of a facepiece respirator. At such a high speed, the contribution given to particle entrapment by diffusion, propelled by Brownian motion, is lost; the high kinetic energy of the flow also negatively impacts interception entrapment principles and the filter efficiency significantly decreases (5–8).

**Figure 1:**
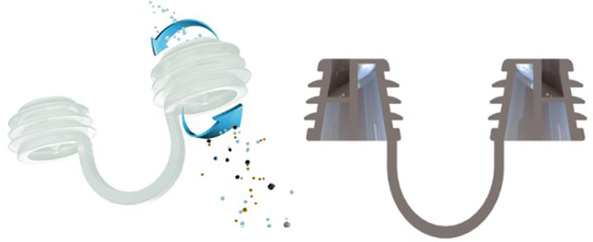
Nasal device under test.

The filter tested in this study (Figure 1) is distributed with the commercial name Sanispira (HSD, Italy) and operates on an aerodynamic filtration principle.

Aerodynamic filters are mechanical filters in which the incoming airflow is disrupted by internal filter surfaces with the aim of generating a difference in the state of motion between the airflow and the transported particles, preparatory to their isolation and collection. Since they do not require a tight mesh of fibers to perform capture, they promise in principle both filtration and limited breathing resistance.

**Figure 2:**
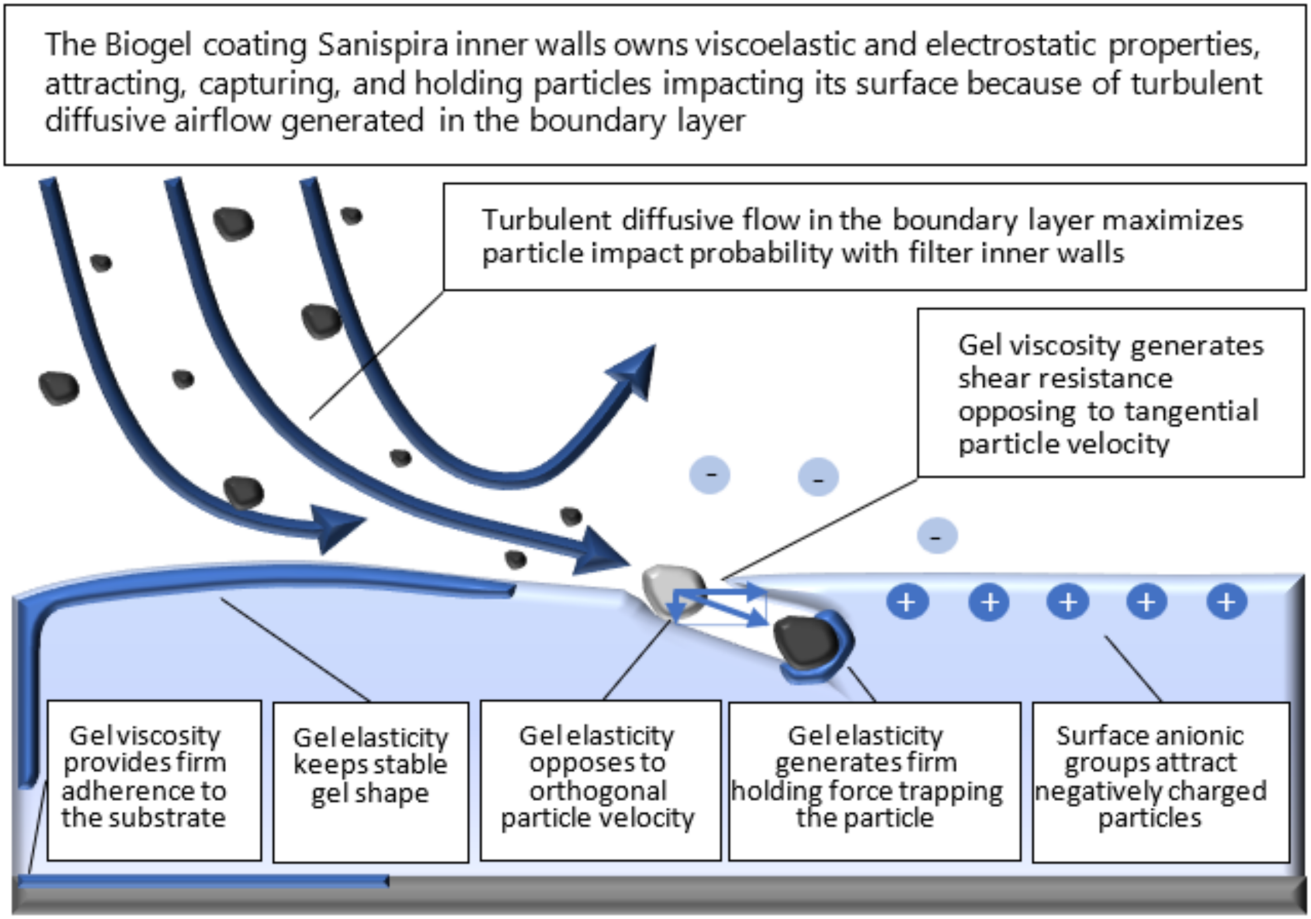
Conceptual representation of the interaction between the airflow and the filter (source: filter manufacturer).

The filter consists of two cones of soft material that are inserted into the nostrils, with soft, thin, outer rings that guarantee maintenance in place. The inside of the cone has a helical shape and is coated with a viscous biogel. According to the manufacturer, the airflow containing the particles is forced by the helical duct to take a high curvature laminar motion thickening near the walls, which maximizes edge effects and supposedly generates a diffusive boundary layer, forcing particles to impact walls and become trapped (Figure 2).

The novelty of this study is testing fine and ultrafine particles with a novel filter based on an aerodynamic filtration principle, totally different from mesh filters, allowing the filter to be as small as a nostril while introducing minimal pressure drop, usable for a long-time in everyday life; in order to investigate such potential use, the test system has been designed to also measure properties such as time of filter saturation and sensitivity to environmental moisture.

## 2. Materials and methods

The NIOSH (National Institute for Occupational Safety and Health) protocol for the “Determination of Particulate Filter Efficiency Level for N95 Series Filters Against Solid Particulates for Non-Powered, Air-Purifying Respirators” uses a fixed flow rate of 85 LPM ± 4 LPM (9, 10). This mode of testing may not be suitable to test a novel gel-coated endonasal device that has a central cavity, and the fluid dynamics have been designed for the respiratory flows of a person at rest or in light physical activity.

Given the novel mode of action and design, these devices shall not fit into the NIOSH mode of testing. Theoretically, 0.3 μm is the most penetrating particle size for pure mechanical filters. Nevertheless, NIOSH uses a challenge aerosol with a count median diameter of 0.075 μm when testing electrostatically charged respirators.

Test methods have been devised in this study aiming at characterizing a device intended for widespread use for the general population, during normal activities in a steady or low-rate state of motion. The target environment is standard (home, office, etc.), where no particle overload is expected, a case which remains outside the scope of a nasal filter. For this reason, tests have been focused on physiological cycling breathing and penetration tests have not been considered.

An alternative test set up was developed to test the endonasal devices' efficiency at a nominal flow rate of ∼6–7 LPM, like that of normal breathing (11).

**Figure 3:**
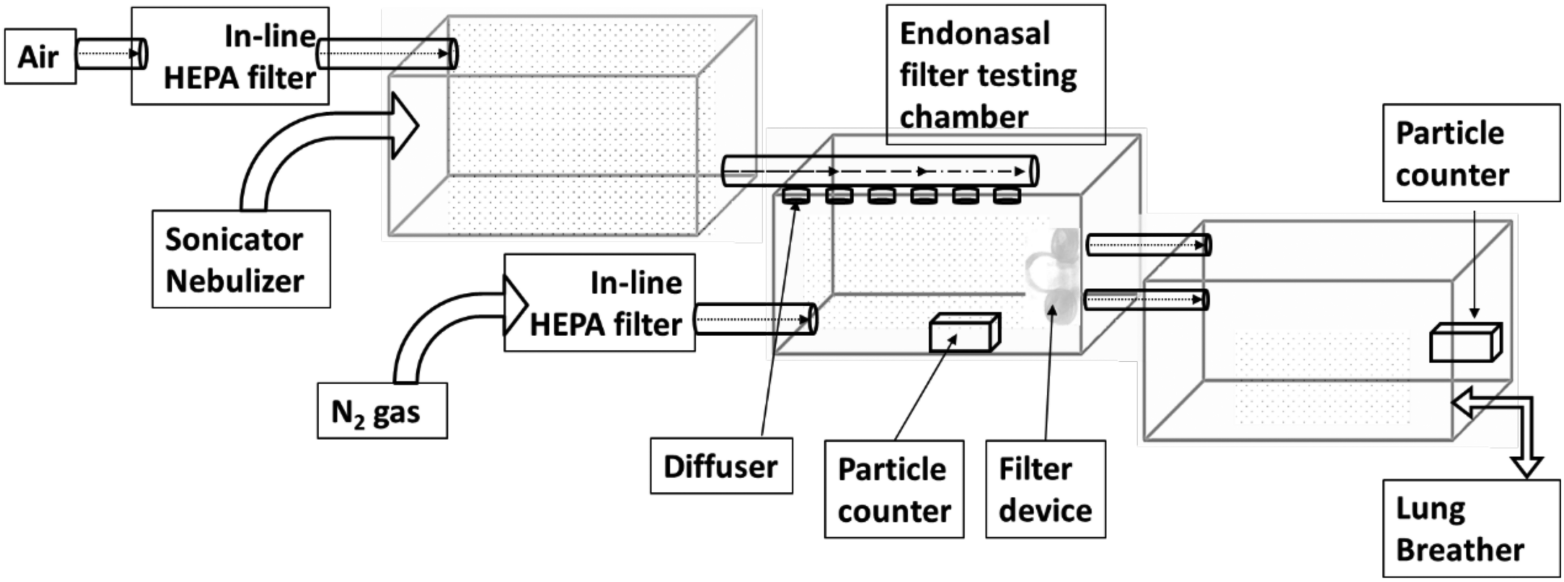
Test system layout.

The aerosol test system has been constructed, composed of three chambers (shown in Figure 3). Each chamber measures 51.5 cm L × 30.8cm W × 32.5 cm H.

Particle sizes of interest were aerosolized using an ultrasonic nebulizer (OMRON NE-U17) run at 6 L/min flow rate and a nebulization volume of 1 m L/min. The aerosolized particles were then passed through a diffuser for an even spread of particles in the chamber.

Timed pulse of compressed industrial grade nitrogen was used to maintain the relative humidity within the test chamber and was passed in-line HEPA filter units (Hepa-Cap 36). All tests were conducted at room temperature.

The setup includes an artificial Lung Breather (IngMar Medical-Quicklung TM Breather) to better represent the analysis of this device. The breather is used in Apneusis mode at 16 BPM with a volume of 500 ml to maximize the air drawn during the inhalation phase and maintain equilibrium in the test chamber.

The test runs followed the maximum loading of 200 mg/m^3^ over a ten-day exposure period with the assumption of a worker exposed to unhealthy air containing 2 mg/m^3^ particulate air over an eight-hour day and an average breathing volume of 10 m^3^ of air. The tests were conducted subjecting the endonasal devices to a low humidity range of 30%–38% and a high humidity range of 70%–95%. All experiments were conducted at room temperature wherein the lowest recording was observed to be 22.7°C and highest was 24.7°C, with an average ± SD of 23.7°C ± 0.7°C. Challenge particle sizes included in the test were 0.5, 1, and 5 μm in diameter. The selected range of monodisperse polystyrene latex spheres (PSL) typically represents biological contaminants including allergens e.g., dust, pet dander, bacteria, mold spores, and smoke particles. We investigated the filtration efficiency of endonasal filters subjected to a 200 mg of extreme particulate matter (PM) loading environment.

Monodisperse particles have been used because the filtration principle of the endonasal aerodynamic device under investigation is totally novel and its behavior towards the capture of particles of different sizes is unknown; this is a fundamental feature, which is well known in the case of mesh filters. Monodisperse particles allow the characterization of the filtration performance vs. specific particle sizes.

The sampling of air particulate count was performed using a SOLAIR 300 counter. The sampling duration was set for 15 s to analyze a volume of ∼7 liters of air, equivalent to an average total lung capacity of an adult human. Sampling was conducted at intervals of 15 min over a period of 1 h. Instantaneous filtration efficiency at fixed time intervals was obtained by confronting particle concentration in the last chamber before and after removing the test filter for the time needed for sampling.

Calibration was done at a measured flow for 1 min, i.e., 28.3 liters volume and at 42.0% RH and 22°C Room Temperature.

All experiments have been performed at approx. 22°C room temperature.

The device is marketed in three sizes (S, M, L), to match users' noses. The diameter of inlet section is: size S (6.44 mm); size M (8,00 mm); and size L (8.90 mm).

Three samples of each size were subjected to the test in a sequential manner.

The experimental setup for pressure drop analyses was made of the following equipment: NXP differential pressure drop sensor model MPX5010DP, Key Instruments air flow meter (up 140LPM) model 2530A4A72BVBN, and a custom case for housing the filter.

## 3. Results

### 3.1. Filtration efficiency

The results of the measurements are presented by their means and standard deviations (see Table 1). Peak particle filtration efficiency >90% was achieved at both low and high humidity for 5 μm, 1 μm, and 0.5 μm challenge particles.

**Table 1:** Filtration efficiencies of endonasal device tested at low (30%–38%) and high (70%–95%) relative humidity under accelerated saturation. Values are expressed in mean ± SD.

Device size	Relative humidity	Particle size [μm]	Sampling time points [min]			
			15	30	45	60
L	Low Humidity	0.5	94.76 ± 1.52	86.22 ± 2.15	65.68 ± 4.77	52.58 ± 3.76
		1	97.91 ± 1.53	92.28 ± 2.10	86.46 ± 4.12	80.01 ± 4.59
		5	99.03 ± 1.43	96.43 ± 1.47	95.30 ± 0.92	92.41 ± 1.60
	High Humidity	0.5	92.56 ± 2.90	84.66 ± 1.06	61.91 ± 5.42	50.87 ± 4.48
		1	97.32 ± 2.07	93.35 ± 2.36	82.72 ± 3.84	73.38 ± 5.28
		5	98.76 ± 1.17	95.53 ± 4.12	92.93 ± 4.02	81.26 ± 2.41
M	Low Humidity	0.5	92.58 ± 0.88	84.45 ± 2.07	64.81 ± 4.14	51.70 ± 4.97
		1	96.11 ± 1.86	92.29 ± 2.11	81.23 ± 3.38	72.56 ± 2.72
		5	98.62 ± 1.32	95.92 ± 1.43	94.50 ± 1.62	89.12 ± 1.63
	High Humidity	0.5	91.37 ± 1.43	83.12 ± 1.85	64.29 ± 2.60	51.86 ± 2.68
		1	95.78 ± 0.92	93.17 ± 1.72	83.03 ± 3.22	72.47 ± 2.98
		5	98.48 ± 1.10	96.20 ± 2.16	94.41 ± 3.23	82.90 ± 3.32
S	Low Humidity	0.5	94.59 ± 1.44	86.79 ± 3.31	65.09 ± 3.28	53.80 ± 3.46
		1	96.69 ± 0.64	92.86 ± 1.38	86.62 ± 2.97	77.78 ± 3.81
		5	99.30 ± 1.12	97.23 ± 1.43	95.31 ± 1.47	90.96 ± 1.71
	High Humidity	0.5	92.91 ± 1.78	85.20 ± 3.52	63.23 ± 4.18	52.11 ± 2.40
		1	95.81 ± 1.11	92.11 ± 1.92	82.16 ± 3.32	73.25 ± 3.05
		5	99.13 ± 0.75	96.59 ± 1.70	93.93 ± 2.58	82.39 ± 3.06

**Figure 4:**
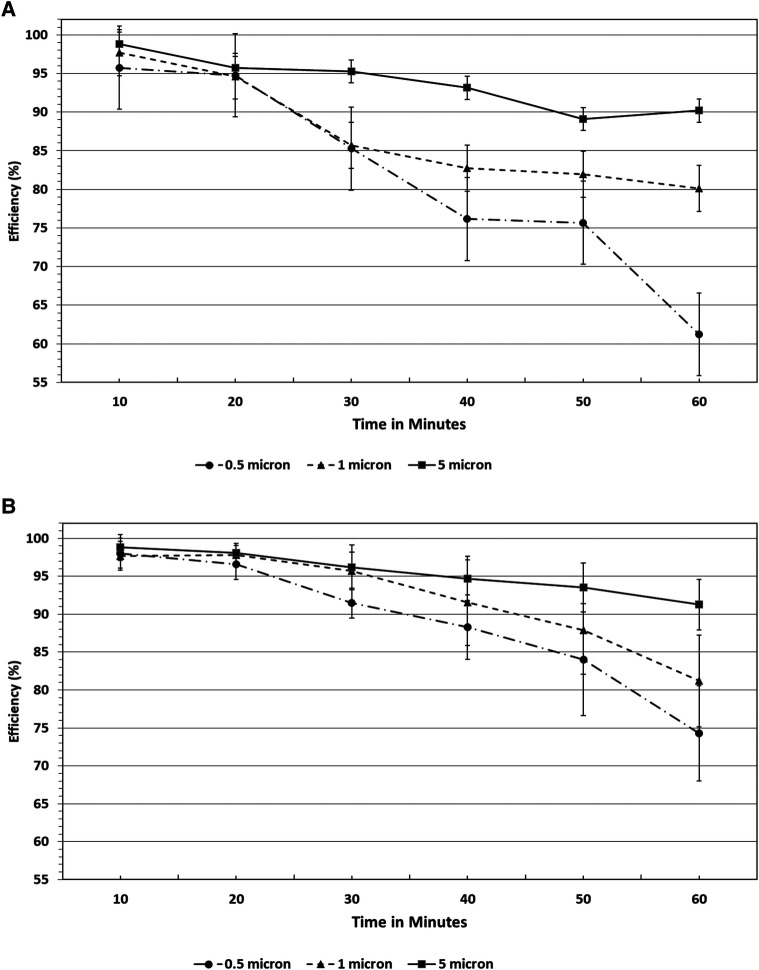
(**A**) Filtration efficiencies of endonasal device tested at high (70%–95%) relative humidity under accelerated saturation. Values are expressed in mean ± SD. (**B**) Filtration efficiencies of endonasal device tested at low (30%–38%) relative humidity under accelerated saturation. Values are expressed in mean ± SD.

Filtration efficiency remained >85% throughout the 60 min test duration with accelerated saturation of 5 μm particles (see Figures 4A,B) under low and high humidity environments. However, <2.0 μm particle filtration efficiency dropped after the 50 min test duration to less than 85%. In particular, the filtration efficiency of 0.5 μm particles were comparatively lower and affected by a low humidity environment past the 30 min exposure period (Figure 4B). Considering the accelerated loading dose of 200 mg/m3, 1 h duration is equivalent to a 10-day exposure level. The nasal filters have demonstrated particle filtration efficiency of ≥85% for particles <2.0 μm size over a 30 min test duration, which is equivalent to a 5-day exposure level. However, the endonasal filters are recommended for daily use akin to filter masks and ensure higher protection level from exposure to these particle ranges. We have not tested real biological aerosols in the present study.

**Figure 5:**
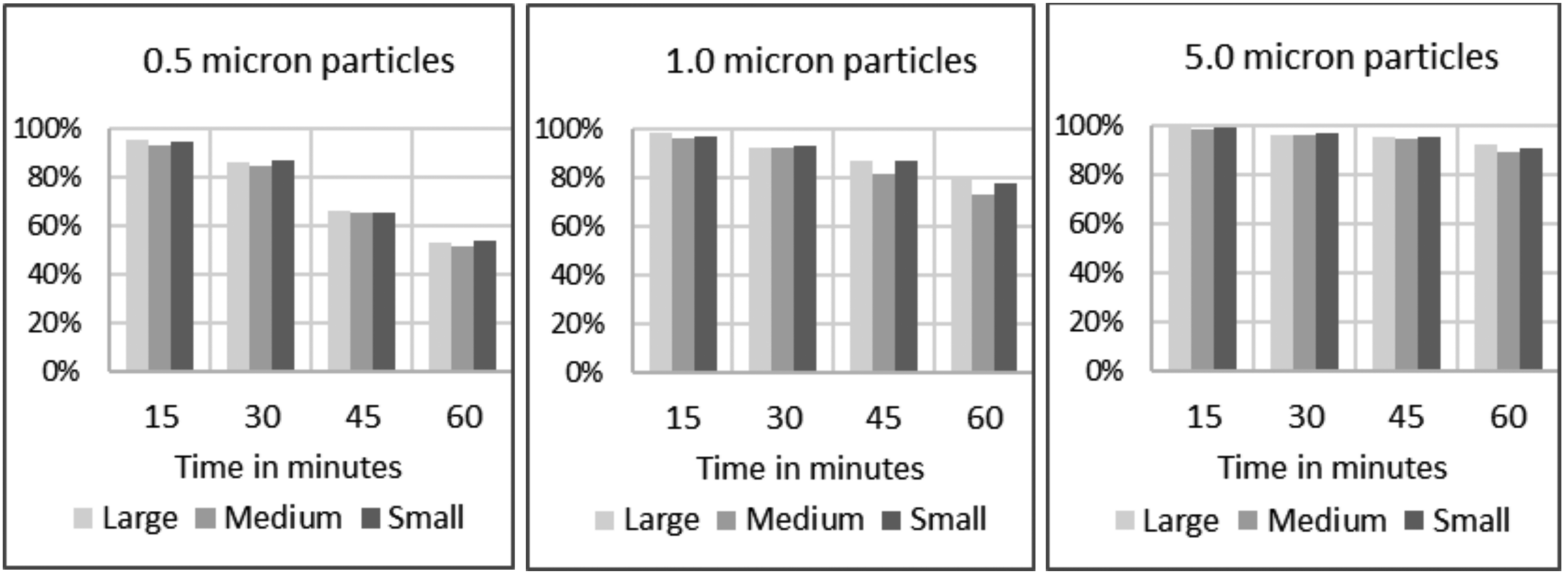
Filtration efficiencies of endonasal device tested at low (30%–38%) relative humidity under accelerated saturation, per size of challenge particles and per size of device under testing.

There is no significant difference observed in the particle filtration efficiency among the three sizes of endonasal filters (S, M, and L) tested, as reported in Figure 5.

### 3.2. Breathing resistance

Pressure drop has been measured with a differential manometer at the flow rate of 6 L/min, i.e., 3 L/min per nostril; results are reported in Table 2.

**Table 2:** Pressure drop of the endonasal filter @ 6l/min flow rate.

Device size	Pressure drop [cmH_2_O]
Size L	0.112
Size M	0.224
Size S	0.377

There are no standardized indexes placing a limit on respiratory resistance for a nasal filter. It is advisable to identify limits based on the specific physiology of nasal respiration.

An index has been proposed (12) that can be obtained by observing that the incidence of clinically observable oral respiration is higher among subjects with a nasal resistance greater than 4.5 cmH_2_O/l/s (77%) compared to those with a nasal resistance lower than this value (26%) (13, 14).

This index R_f_ is specifically adherent to the respiratory physiology of the nose and is proposed as a reference for deriving an applicable index to characterize breathing resistance associated to endonasal devices.

$$\;\;\;\;R_{f}=\frac{Pressure\;Drop\;[cm\;H_{2}O]}{Flow\;rate\;\left[\frac{l}{s}\right]}\leq 4,5\;\frac{cm\;H_{2}O}{\frac{l}{s}}$$

The values of the R_f_ index for the three sizes, L, M, and S, are reported in Table 3.

**Table 3:** *R_f_* index calculated per each device size.

Device size	Rf [cmH_2_O/L/s]
Size L	1.12
Size M	2.24
Size S	3.77

### 3.3. Quality factor

A Quality Factor has been proposed to evaluate the overall performance of a filtering system through an index based on knowledge of filtration efficiency and respiratory resistance (7, 16).

$$Q_{f}=-\frac{\ln(1-F_{e}\text{\%})}{R_{f}}$$

The higher the index, the better the performance.

The Q_f_ index has been calculated for the size L filter at one time instant (Table 4).

**Table 4:** Quality factor for size L filter per particle size.

Particle size [μm]	Q_f _× 10^−4^ [Pa^−1^]
0.5@15 min	2,681
1@15 min	3,512
5@15 min	4,214

The Q_f_ of the size L filter challenged by 0.5 μm particles is 2,681 × 10^−4^ [Pa^−1^].

For reference, the Q_f_ of a an N95-B40 certified facepiece respirator equals 3,210 × 10^−4^ [Pa^−1^], when challenged by an NaCl challenge with a mean value of 0.6 μm; a nasal filter from an unknown manufacturer scores 22.6 × 10^−4^ [Pa^−1^] when challenged by NaCl particles (11).

## 4. Discussion

A nasal filter shows potential attractive properties to protect the general population in day-to-day life: it does not affect speaking and hinder relations; is almost invisible; can be more tolerable than filtering facepieces, which can increase humidity; and; it can be used for a long-time, thereby extending protection to further contexts (e.g., eating).

Efficacy is expected to be lower than facepieces since in-nose filtration is extremely demanding for size constraints and out of the recognized performance envelope allowing fibrous filters to perform fine and ultra-fine filtration. Nevertheless, devices should be evaluated in terms of their effectiveness, i.e., the capacity to protect the wearer over the course of a full day while carrying out typical activities, also limiting problems of low compliance and misuse of facepieces. Airborne pathogens are mainly contracted through respiratory pathways, especially through the nose, since airborne particles are mostly filtered within the nasal airway (16). The important role played by the nasal epithelia in COVID-19 has been further magnified after the emergence of the many Omicron sublineages, which exhibit highly efficient replication within the nasal mucosa; furthermore, Angiotensin converting enzyme 2 (ACE2) and trans-membrane protease serine 2 (TMPRSS2), the primary entry factors of SARS-CoV-2, are expressed in a wide range of human mucosal surfaces of the head and neck, most prominently in the nasal cavity and trachea (17).

Recently we described one operative protocol for testing the efficacy of nasal filters in preventing airborne transmission of respiratory infectious agents (18). Our setting is a simulation of a real-life situation and the absolute efficacy of endonasal filters can only be tested live. However, the main limit of our study is related to the controlled laboratory condition tested and to the translatability of the results in a real setting, in terms of virion numbers that are able to infect the host and so produce illness.

Another study has reported higher filtration efficiency of fine particles with electrospun nanofiber filters in comparison to three commercially available nasal filters using monodisperse polystyrene latex (PSL) particles of fine (<2.5 μm) and ultra-fine (<0.1 μm) size range without a significant pressure drop for up to 4 h (19). Recently, a novel bioinspired 3D-printed filter design with five levels of tortuous airflow channels was shown to have a low pressure drop compared to commercial masks with higher capturing efficiency of particles less than 12 μm with >80%, however, it also exhibited lower capturing efficiency with smaller particle diameters and higher velocity of airflow (20).

In the work presented, the tests aimed at characterizing a device intended for widespread use for the general population, during normal activities in steady or low-rate state of motion. The target environment is a standard one (home, office, etc.); for this reason tests have been focused on physiological cycling breathing and penetration tests have not been considered.

The endonasal devices of all three sizes were exposed to loading of 200 mg/m^3^ over a 60 min interval. Achievement of >80% filtration efficiency for PM2.5 particles over a 30 min interval indicates the general limitation of usability in such an environment.

Assuming an individual would breathe about 10 m^3^ of air over an eight-hour day with exposure of 2 mg/m^3^, it would take 10 days of continuous use to reach 200 mg. By following the 3 M time use limitation on P-series filters, the endonasal devices would have to be replaced after no more than 2.5 days (i.e., 16–20 h assuming 8 h working shifts) before any potential decrease in filter efficiency.

Nevertheless, the gel stability upon exposure at low humidity conditions for a 10-day period was not validated in this study and hence we cannot advise the use of the filter device for more than 8 h.

In a recent study by Zhu et al. (11), nasal filters were challenged with sodium chloride aerosol with particle distribution (0.02–1 µm) as a surrogate for PM1 particle penetration levels in different sizes of nasal filters. The study assessed the limitations of the model used to test nasal filters due to the higher penetration rate % observed against the claims that they would have offered a sufficient level of protection. Though in the present study PM1 aerosol particles range were not analyzed over a size range except for 0.5 µm, this could be taken up for future testing of our endonasal devices to assess its filtration efficiency.

Some insight into the tolerability of the device can be inferred from the calculation of the R_f_ index, based on human breathing physiology; results are below limit thresholds, especially for sizes L and M, suggesting that the device delivers filtration at a reduced resistance to flow and is compatible with human nose breathing. Further investigation is necessary to better characterize the interaction with the respiratory system and the nasal mucosa.

Given the original working principle with respect to consolidated fibrous filters, the adoption of an overall Q_f_ quality factor index allows to provide the positioning of the new technology; the ability to deliver a Q_f_ in the same range of a certified filtering facepiece respirator, even if the nasal filter is crossed by a face flow in the order of m/s, suggests that aerodynamic filtration has the disruptive potential to deliver ultra-compact, in-nose devices for fine and ultra-fine filtration.

The proposed test system based on monodispersed particles correlates filtration performance of a given device to a specific range of particles size and, hence, application (e.g., pollens, mites, PM); this is indispensable for ultra-compact endonasal filters running at high face velocities. The extreme performance envelope requires filtration efficiency and breathing resistance tailored for specific applications.

## 5. Conclusions

Making an endonasal filter trapping ultrafine particles and aerosols, nearly invisible and with negligible respiratory resistance, available is a shared target, even more deeply felt after the acute pandemic phase, and towards which many research efforts are concentrating. Measured filtration performance of the endonasal filter under investigation shows that the aerodynamic endonasal filter tested has efficacy in filtering fine and ultra-fine particles <2.5 μm, peaking above 90% across the size spectrum 5.0 ÷ 0.5 μm. This range encompasses particles more injurious to the lungs as well as droplet and droplet nuclei emitted by humans (21–24), potentially capable to transmit pathogens, such as SARS-COV-2.

Performance remains substantially unchanged at different device sizes and relative humidity conditions. Although filter saturation occurs under accelerated testing, after 10 days equivalent of use, gel stability for a 10-day period was not validated and its use is not advisable for longer than 8 h.

Filtration performance is available to the user at an acceptable resistance to flow, as shown by the R_f_ index which was measured below the threshold of 4.5 cmH_2_O/L/s for the three sizes tested, expected to be compatible with continuous nasal breathing at rest or limited physical activity.

The overall quality index Q_f_ confirmed that the proposed aerodynamic filtration performs very advantageously in size-constrained filtration applications, where it can provide fine and ultra-fine filtration despite high face velocities with a quality index comparable to an N90 face respirator.

Overall results appear well beyond the current state of the art concerning compact endonasal filters and suggest that the filter can protect the population in everyday life from risks associated with inhalable fine and ultra-fine particles, introducing a disruptive personal filtration technology joining together high compactness, ultrafine particle filtration, and limited resistance to flow, enabling for the first time in-nose ultrafine particle filtration. Such a performance envelope is unthinkable with conventional mesh filters and paves the way to a totally new class of devices, destined for everyday life.

Further investigation is suggested to extend knowledge about filter behavior over time and in different conditions, such as ambient temperature, to evaluate the proprietary nasal gel filtration performance at high/low temperatures, as well to validate clinical tolerability and acceptability by users.

## Data Availability

The original contributions presented in the study are included in the article/supplementary files, further inquiries can be directed to the corresponding author/s.

## References

[1] NIOSH. Health hazard evaluation report: NIOSH investigation of 3M model 8000 filtering facepiece respirators requested by the California occupational safety and health administration, division of occupational safety and health, Oakland, CA. By Berry Ann R. Pittsburgh, PA: U.S. Department of Health and Human Services, Centers for Disease Control and Prevention, National Institute for Occupational Safety and Health, NIOSH HETA No. 2010-0044-3109 (2010).

[2] Medina DE. Filtration performance of a NIOSH-approved N95 filtering facepiece respirator with stapled head straps (2010). Graduate Theses and Dissertations. Available at: http://scholarcommons.usf.edu/etd/1709

[3] Janssen L Bidwell J. Performance of four class 95 electret filters against diesel particulate matter. J Int Soc Respir Prot. (2006) 23:21–9.

[4] Swift DL Proctor DF. Access of air to the respiratory tract. In: Brain JD Proctor DF Reid LM, editors. Respiratory defence mechanisms. New York, NY: Marcel Dekker (1977). p. 63–93.

[5] Wang A Richardson AW Hofacre KC. The effect of flow pattern on collection efficiency of respirator filters. J Int Soc Respir Prot. (2012) 29:41–54.

[6] Kumar A Sangeetha DN Yuvaraj R Menaka M Subramanian V Venkatraman B. Quantitative performance analysis of respiratory facemasks using atmospheric and laboratory generated aerosols following with gamma sterilization. Aerosol Air Qual. Res. (2020) 21:200349. 10.4209/aaqr.2020.06.0349

[7] Kumar A Joshi S Venkatesan S Balasubramanian V. Assessment of N95 respirator for reuse after sterilization: filtration efficacy, breathing resistance, quality factor, chemical structure and surface charge density. medRxiv. (2021). 10.1101/2021.05.25.21257801

[8] Eninger RM Honda T Adhikari A Heinonen-Tanski H Reponen T Grinshpun SA. Filter performance of N99 and N95 facepiece respirators against viruses and ultrafine particles. Ann Occup Hyg. (2008) 52(5):385–96. 10.1093/annhyg/men019 18477653 PMC6768072

[9] NIOSH. Procedure No. TEB-APR-STP-0059. Determination of particulate filterfilter efficiency level for N95 series filters against solid particulates for non-powered, air purifying respirators standard testing procedure (STP). Rev 3.0 (2016).

[10] Gao S Kim J Yermakov M Elmashae Y He X Reponen T Penetration of combustion aerosol particles through filters of NIOSH-certified filtering facepiece res-pirators (FFRs). J Occup Environ Hyg. (2015) 12(10):678–85. 10.1080/15459624.2015.1043057 26010982 PMC6750198

[11] Pleil JD Ariel Geer Wallace M Davis MD Matty CM. The physics of human breathing: flow, timing, volume, and pressure parameters for normal, on-demand, and ventilator respiration. J Breath Res. (2021) 15(4):10.1088/1752–7163/ac2589. 10.1088/1752-7163/ac2589 PMC8672270 34507310

[12] Zhua J Jinga P Hao Y He X Wang L Zhang R Performance of a new model of nasal filter when challenged against PM1 aerosols. Environ Pollut Bioavailab. (2021) 33(1):388–94. 10.1080/26395940.2021.1991847

[13] Watson RM Warren DW Fischer ND. Nasal resistance, skeletal classification, and mouth breathing in orthodontic patients. Am J Orthod. (1968) 54(5):367–79. 10.1016/0002-9416(68)90305-9 5238999

[14] Warren DW Hinton VA Pillsbury HC 3rd Hairfield WM. Effects of size of the na sal airway on nasal airflow rate. Arch Otolaryngol Head Neck Surg. (1987) 113(4):405–8. 10.1001/archotol.1987.01860040067019 3814392

[15] Hinds WC. Respiratory deposition in aerosol technology: filtration, properties, behaviour, and measurement of airborne particles. Vol. 11. 2nd ed. New York, America: John Wiley & Sons, Inc, Publisher (1999). 182–205.

[16] Nocini R Henry BM Mattiuzzi C Lippi G. Improving nasal protection for preventing SARS-CoV-2 infection. Biomedicines. (2022) 10(11):2966. 10.3390/biomedicines10112966 36428534 PMC9687306

[17] Wu CT Lidsky PV Xiao Y Cheng R Lee IT Nakayama T SARS-CoV-2 replication in airway epithelia requires motile cilia and microvillar reprogramming. Cell. (2023) 186(1):112–130.e20. 10.1016/j.cell.2022.11.030 36580912 PMC9715480

[18] Semeraro S Gaetano AS Zupin L Poloni C Merlach E Greco E Operative protocol for testing the efficacy of nasal filters in preventing airborne transmission of SARS-CoV-2. Int J Environ Res Public Health. (2022) 19:13790. 10.3390/ijerph192113790 36360670 PMC9654745

[19] Han TT Yang L Lee KB Mainelis G. Design and development of a novel nanofiber nasal filter (NNF) to improve respiratory health. Aerosol Air Qual. Res. (2018) 18:2064–76. 10.4209/aaqr.2018.03.0086

[20] Yuk J Chakraborty A Cheng S Chung C-I Jorgensen A Basu S On the design of particle filters inspired by animal noses. J R Soc Interface. (2022) 19:20210849. 10.1098/rsif.2021.0849 35232280 PMC8889202

[21] Stadnytskyi V Bax CE Bax A Anfinrud P. The airborne lifetime of small speech droplets and their potential importance in SARS-CoV-2 transmission. Proc Natl Acad Sci USA. (2020) 117(22):11875–7. 10.1073/pnas.2006874117 32404416 PMC7275719

[22] Morawska L Cao J. Airborne transmission of SARS-CoV-2: the world should face the reality. Environ Int. (2020) 139:105730. 10.1016/j.envint.2020.105730 32294574 PMC7151430

[23] Jayaweera M Perera H Gunawardana B Manatunge J. Transmission of COVID-19 virus by droplets and aerosols: a critical review on the unresolved dichotomy. Environ Res. (2020) 188:109819. 10.1016/j.envres.2020.109819 32569870 PMC7293495

[24] Wang CC Prather KA Sznitman J Jimenez JL Lakdawala SS Tufekci Z Airborne transmission of respiratory viruses. Science. (2021) 373(6558):eabd9149. 10.1126/science.abd9149 34446582 PMC8721651

